# Winning the race with aging: age-related changes in gait speed and its association with cognitive performance in dogs

**DOI:** 10.3389/fvets.2023.1150590

**Published:** 2023-06-15

**Authors:** Alejandra Mondino, Michael Khan, Beth Case, Gilad Fefer, Wojciech K. Panek, Margaret E. Gruen, Natasha J. Olby

**Affiliations:** Department of Clinical Sciences, College of Veterinary Medicine, North Carolina State University, Raleigh, NC, United States

**Keywords:** canine cognitive dysfunction syndrome, canine gerontology, mobility, walking speed, memory, attention

## Abstract

**Introduction:**

In humans, gait speed is a crucial component in geriatric evaluation since decreasing speed can be a harbinger of cognitive decline and dementia. Aging companion dogs can suffer from age-related mobility impairment, cognitive decline and dementia known as canine cognitive dysfunction syndrome. We hypothesized that there would be an association between gait speed and cognition in aging dogs.

**Methods:**

We measured gait speed on and off leash in 46 adult and 49 senior dogs. Cognitive performance in senior dogs was assessed by means of the Canine Dementia Scale and a battery of cognitive tests.

**Results:**

We demonstrated that dogs' food-motivated gait speed off leash is correlated with fractional lifespan and cognitive performance in dogs, particularly in the domains of attention and working memory.

**Discussion:**

Food-motivated gait speed off leash represents a relatively easy variable to measure in clinical settings. Moreover, it proves to be a more effective indicator of age-related deterioration and cognitive decline than gait speed on leash.

## Introduction

Canine geriatric syndrome has been proposed as a new concept that describes “the constellation of key physical, functional, and metabolic changes that characterize aging in dogs and predispose to age-related dysfunction, disease, and death” (1). Some of the most remarkable age-related changes associated with this syndrome occur in motor and cognitive function (1, 2). Physical impairments and slowing of movement reflect the diminished performance of numerous systems, such as musculoskeletal system, central and peripheral nervous system, and sensory organs (3–5).

In humans, gait speed is a valid, reliable, and simple measure of physical function. This is considered a crucial component of geriatric evaluation because it is associated with disability, hospitalization, mortality and cognitive decline (6–9). In fact, gait speed is considered a “sixth vital sign” in older adults together with blood pressure, respiration, pulse, temperature, and pain (7). The link between gait speed and cognition has become particularly interesting since it has been demonstrated that slow gait speed can be a harbinger of cognitive decline and dementia (10–12).

Dogs are an excellent animal model for studying aging (13) because they suffer from age-related impairments in mobility (14, 15) and cognition (16, 17) and may develop canine cognitive dysfunction syndrome (CCDS) which has several similarities with human Alzheimer's disease (18). Therefore, a similar association between gait speed and cognitive performance is expected in dogs as in humans. Age-related changes in gait speed do happen in dogs; Morgan et al. (15) showed a weak negative correlation between age and gait speed. They found that senior dogs (older than their 75% of their expected lifespan) can be up to 63% slower than younger dogs (15). However, these authors did not evaluate whether there is an association between gait speed and cognitive performance.

In dogs, cognitive performance can be assessed in several ways. Owner-based questionnaires such as the Canine Dementia Scale (CADES) have undergone psychometric validation (19) and represent an accessible way to detect the occurrence of behaviors associated with CCDS. Additionally, our group has recently shown the feasibility and utility of a battery of cognitive tests in senior dogs. These tests allow us to evaluate different domains of cognition such as sustained attention, memory, executive function and response to social cues (16).

In this study, we first investigated age-related changes in gait speed on and off leash in adult and senior dogs. Secondly, in the group of senior dogs, we evaluated the association between gait speed and cognitive performance (evaluated by CADES questionnaire and the cognitive tests battery) as well as the role of pain in gait speed.

## Materials and methods

### Study population

All procedures were approved by the North Carolina State University (NCSU) Institutional Animal Care and Use Committee, protocol number: 21–303 and 21–306. Two groups of client-owned dogs were included in this study, a group of senior dogs (older than 75% than their expected lifespan) and a group of adult dogs (older than 1 year and younger than 75% of their expected lifespan)(20). Since expected lifespan in dogs is highly associated with the dogs' body size, we calculated it for each individual dog using the formula proposed by Greer et al. (21) which takes into consideration dogs' height and weight. The group of senior dogs are a population of dogs participating in a longitudinal study of neuro-aging at the North Carolina State University (NCSU) College of Veterinary Medicine [described elsewhere (16, 22, 23)]. To be included, dogs had to be free of co-morbidities that could impede their ability to perform the tests such as inability to walk or blindness. The dogs' most recent visit in the study was used for this cross-sectional analysis. The group of adult dogs was recruited through emails and flyers sent to the NCSU community to establish gait speed in a younger cohort of dogs. We selected dogs from a range of different breeds, sizes, and ages. Dogs with abnormal orthopedic or neurological examination and blind dogs were excluded. Owners dropped the dogs off in the morning and picked them up in the afternoon. In a screening visit, the dogs' behavior toward the experimenters was evaluated by bringing the dogs in the cognitive testing room and offering them treats. Dogs that displayed signs of fear, aggression or anxiety, or wouldn't approach the experimenters to get treats (or showed no food motivation), were not included in the study. We collected demographic information for every dog and health status was evaluated by veterinarians (AM, GF, WP, and NO). We performed physical, orthopedic, and neurological examinations. During physical examination, height to the dorsal border of the scapula (withers) was measured. In the population of senior dogs, urinalysis, biochemistry panel and complete blood cell count were also performed to identify co-morbidities. Dogs with active, severe disease processes that could affect their ability to participate in the longitudinal study of Neuro-Aging were excluded.

### Gait speed evaluation

On leash and off leash gait speed in dogs was determined by performing 3 consecutive trials and calculating the average speed. For on leash gait speed, each trial consisted of walking the dog on their own leash over a 5 m long indoor flat course. A stopwatch was started when the dog crossed the start line and stopped when it crossed the finish line with one of the thoracic limbs. The experimenter let the dog set the pace and was careful not to put pressure on the leash, the leash was always held loosely (Supplementary Video S1). For the off leash trials, a handler held the dog at the starting line of the 5-meter course. The experimenter walked to the dog and showed the dog a treat, then the experimenter stood at the finish line, called the dog by their name and said “Look”. The handler said “Ready, set, go” and released the dog on “Go” (Supplementary Video S2). At the same time the experimenter started the stopwatch. The stopwatch was stopped when the dog crossed the finish line with one of their thoracic limbs. If in any of the trials (either on or off leash) the dog was distracted and stopped walking, the trial was discarded and repeated. The average of the time recorded for each test was divided by 5 to calculate the speed in m/s.

### Cognitive evaluation in senior dogs

Behavioral changes associated with cognitive dysfunction syndrome were assessed by a validated clinical metrology instrument, the Canine Dementia Scale (CADES) sent to owners to complete on-line. The first time the owners completed the questionnaires in the Longitudinal Study of Neuro-Aging the questionnaires were reviewed with the owners to ensure they understood the questions. Owners were encouraged to reach out to the investigators if they had any questions about the completion of the questionnaire. This questionnaire asks the owners about the frequency of behaviors related to spatial disorientation, social interactions, sleep-wakefulness cycle and house soiling (19). The score range for this questionnaire goes from 0 (completely normal) to 95 (severely affected) and allows classification of dogs into 4 different categories: normal (0–7), mild cognitive impairment (MiCI, 8–23), moderate cognitive impairment (MoCI, 24–44) and severe cognitive impairment (SCI, 45–95).

Cognitive testing was also performed as described by Fefer et al. (16) to evaluate attention (sustained gaze test), social cues (pointing test), working memory, executive control (inhibitory control cylinder task) and problem solving (detour cylinder task). The sustained gaze test measures the time the dog holds the gaze with the experimenter holding a treat near their face. The pointing test determines the percentage of correct trials in which dogs can retrieve a treat in a two-choice task when the experimenter points to where it was hidden. For the working memory task, the dog is allowed to see where the experimenter hides a treat in a two-choice task and the upper threshold of time that dogs are able to remember and select where the treat was hidden is measured. In inhibitory control, test dogs are asked to retrieve a food reward from a transparent cylinder open on both ends without touching the cylinder. The detour task is similar but adds an additional difficulty–the dog's preferred side for entering the cylinder is covered, and the dog needs to retrieve the treat using the opposite side. The percent of correct trials was calculated for these two tasks. Tests were performed in the morning, between 8 AM and 12 PM, in a quiet room designated for this type of study. Dogs were provided with short breaks when they seemed tired or to be losing interest (failing to make choices in the tasks). Pre-determined abort criteria were used to terminate testing if dogs were not making choices during the tasks after the breaks.

### Pain evaluation in senior dogs

We evaluated pain in the population of senior dogs to determine its relationship with gait speed.

Owners' perception of dogs' pain was assessed by means of the Canine Brief Pain Inventory (CBPI) questionnaire. This questionnaire can be divided into two subscales, a pain severity subscale (which assesses the severity of the dog's pain according to the owners) and a pain interference subscale (which assesses how pain interferes with dog's activities) (24). As with CADES questionnaire, owners underwent initial training on the questionnaire and were encouraged to ask questions if further clarification was needed. Additionally, we scored joint pain during orthopedic examination. Each appendicular joint was assessed, with all toes considered as one single site for each paw, so the joints evaluated were manus, carpus, elbow, shoulder, pes, hock, stifle and hip. Joint pain was quantified by scoring each joint from 0 to 4 during flexion and extension as follows: 0: Does not notice; 1: Orients to site, does not resist or mild resistance; 2: Orients to site, slight objection to manipulation; 3: Withdraws from manipulation, may vocalize, may turn to guard area; and 4: Tries to escape/prevent manipulation, may bite or show aggression. Total joint pain was determined as the sum of the score in each joint on a scale of 0–64 (25).

### Statistical analysis

Statistical analyses were performed using JMP Pro, Version 16.0 SAS Institute Inc., Cary, NC and IBM SPSS Statistics, Version 27.0. IBM Corp. Armonk, NY. Normality was assessed in all variables by means of Shapiro-Wilk test. Variables that were not normally distributed were presented as median (range), while normally distributed variables were presented as mean ± standard deviation. As most variables were not normally distributed, correlation between two variables was assessed using the non-parametric Spearman's ρ test. For each group of dogs, we assessed the potential impact of height on gait speed by calculating the correlation between gait speed on leash and gait speed off leash with the height of the dogs. In the group of senior dogs, we tested whether there was a correlation between gait speed on leash and gait speed off leash (independent variables) with CADES scores and each cognitive test result (dependent variables). We have demonstrated that CADES scores and performance in cognitive tests are correlated with fractional lifespan, with older dogs having more cognitive impairment than younger dogs. Therefore, for variables that were associated with fractional lifespan, fractional lifespan was included as a covariate in multivariate analyses. For these analyses, variables that were not normally distributed were transformed by log(x+1). We also evaluated, in the group of senior dogs, whether pain was associated with gait speed on and off leash by means of Spearman's correlation analyses. In these analyses we included the two CBPI subscales scores and total joint pain measured by orthopedic examination as independent variables and gait speed on and off leash as dependent variables. Finally, we determined the test-retest reliability comparing the three measurements of both gait speed on and off leash using an intraclass correlation coefficient (ICC) with a two-way random effects, absolute agreement, single rater/measurement, i.e., ICC_(2, 1)_ (26).

Due to the exploratory nature of this study, we did not perform correction for multiple comparisons (27). Results were considered statistically significant if *p* < 0.05. All the collected data are provided in Supplementary File 1.

## Results

### Study population

A total of 109 dogs were screened for this study. Nine dogs were excluded for being too anxious and refusing to approach the experimenter in order to obtain treats. One dog was excluded because he was blind in his left eye and had severe cachexia, one dog was excluded for having antibiotic resistant urinary tract infection, three dogs were excluded for abnormalities in their bloodwork; one had marked liver enzymes (alkaline phosphate, alanine transaminase and aspartate aminotransferase) elevation, one had leukocytosis, and was therefore tested for infectious disease and was positive for Rocky Mountain Spotted Fever and the other one showed electrolyte imbalances (hypocalcemia, hypomagnesemia and hyperkalemia), and had also a history of seizures. Forty six adult dogs [4.7 ± 2.30 years old (Range: 1.04–9.12)] and 49 senior dogs [13.01 ± 1.70 years old (Range: 9.8–16.2] were finally enrolled. Their mean fractional lifespan was 0.38 ± 0.19 (Range 0.08–0.71) and 1.04 ± 0.12 (Range 0.77–1.23) for each group respectively.

Twenty-four of the adult dogs were females (18 spayed) and 22 were males (17 neutered). The most represented breeds were Border collie with 7 dogs, German Shepherd with 6 dogs, and golden retriever and Siberian husky with 5 dogs each. There were 4 each of Labrador retriever, Jack Russell terrier, Maltese terrier and pit bull terrier, 2 Scottish terriers and 1 dog of each of the following breeds: beagle, Boston terrier and chihuahua. The median height of the group of adult dogs was 55 cm (Range 23–78 cm).

Thirty-three of the senior dogs were spayed females and 16 were neutered males. There were eighteen mix breed dogs, 7 Labrador retrievers, 5 pit bull terriers, 3 beagles, 2 golden retrievers and Border collies and one of each of the following breeds: Basset hound, Bernese Mountain dog, Brittany spaniel, Cairn terrier, Pembroke Welsh corgi, dachshund, German shepherd dog, German short-haired pointer, great Dane, Jack Russell terrier, Pomeranian, and Irish setter. The median height of these dogs was 51.53 cm (Range 19.89–89.5 cm). There was no significant difference in height between the adult and senior dogs (*t* = 0.64, *p* = 0.523).

### Speed on and off leash

Gait speed on and off leash were not normally distributed. The median speed for adult and senior dogs on leash was 0.96 m/s (range 0.66–1.90 m/s) and 0.77 m/s (range 0.41–1.46) and the median speed off leash was 2.06 m/s (range 0.83–3.77) and 1.26 m/s (range 0.43–2.84), respectively.

In the group of adult dogs, there was a significant correlation between height and gait speed off leash (ρ = 0.340, *p* = 0.017) but no correlation was found with gait speed on leash (ρ = 0.198, *p* = 0.173). In contrast, speed on leash but not off leash was correlated with height in the group of senior dogs (ρ = 0.367, *p* ≤ 0.009 and ρ = 0.113, *p* = 0.442 respectively).

Gait speed on leash had a moderate test-retest reliability (ICC_(2, 1)_ = 0.72 (0.63–0.79) *p* ≤ 0.001) and gait speed off leash had a good test-retest reliability [ICC_(2, 1)_ = 0.82 (0.74–0.87) *p* ≤ 0.001].

### Effect of age on speed

**Figure 1:**
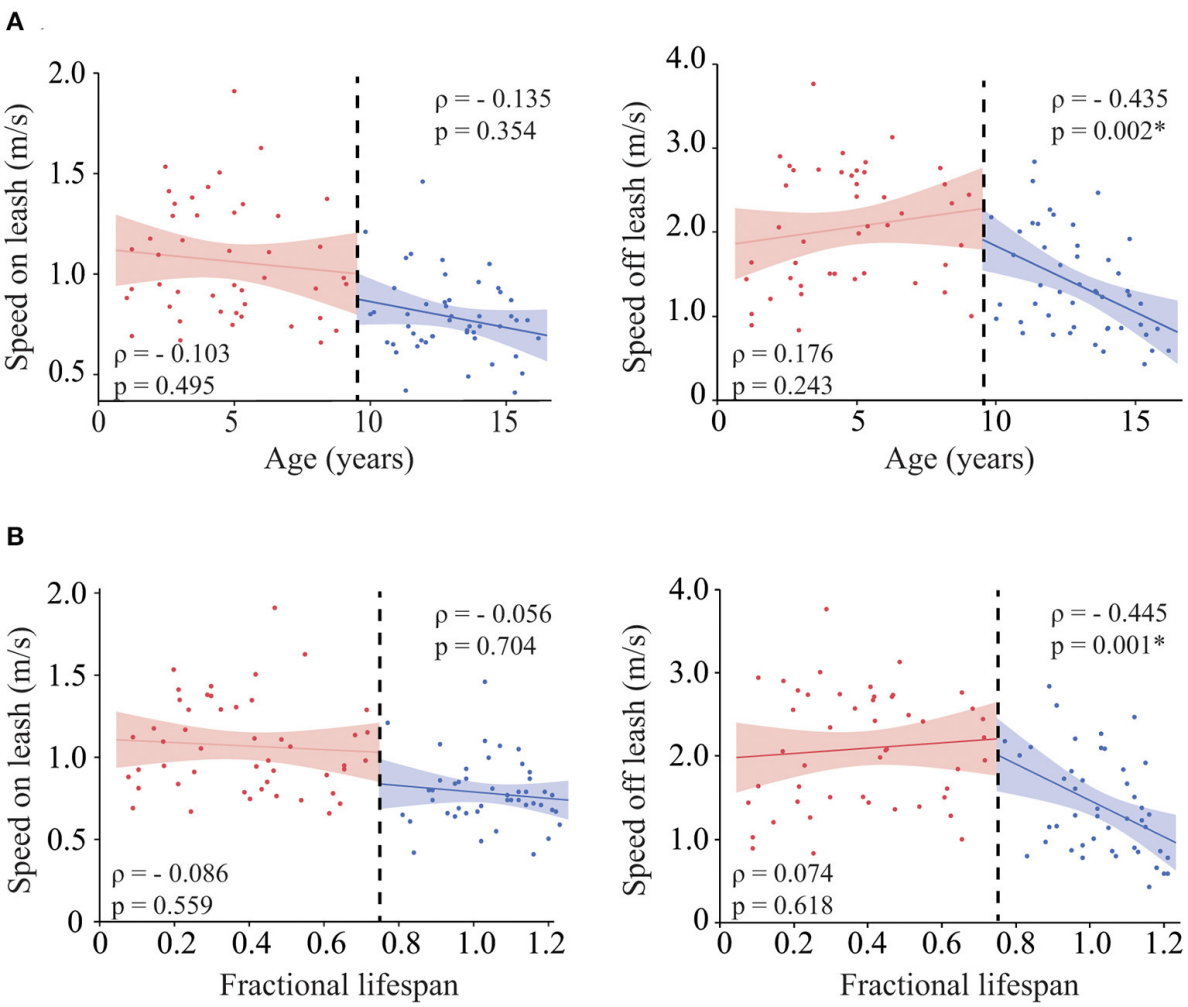
Role of age in gait speed. Correlation between age **(A)** [and fractional lifespan **(B)**] and gait speed on leash (left), gait speed off leash (right). The analyses were performed for each group of dogs (adult vs. senior) independently. For easier visualization both groups are shown in the same graph separated by a black dashed line. Asterisks indicate significant differences.

Both speed on and off leash were negatively correlated with age (ρ = −0.501, *p* ≤ 0.001 and ρ = −0.459, *p* ≤ 0.001, respectively). However, when looking at the scatter plot, the correlation between gait speed off leash and age did not follow a linear trend; speed remained stable up to approximately 12 years and rapidly declined in older dogs. Therefore, we evaluated the role of age in each group of dogs separately (Figure 1A), and we found that while speed on leash was not correlated with age in either adult or senior dogs, speed off leash was negatively correlated with age in the group of senior dogs, but not in adult dogs. Similar results were obtained when looking at age as fractional lifespan (Figure 1B), but the correlation with gait speed off leash was slightly higher than with chronological age. Therefore, fractional lifespan was used instead of chronological age in further analyses.

### Relationship between gait speed and cognition in senior dogs

Senior dogs' median CADES score was 12 (Range 0–74). Fifteen dogs were classified as normal, 14 as having MiCI, 11 as MoCI and 9 as SCI. Not all 49 dogs completed all cognitive tasks; the number of dogs who completed each task was: sustained gaze = 49, working memory = 47, pointing task = 40, inhibitory control task = 48, and detour task = 46. Median sustained gaze time was 18.46 s (Range 0–60) and median memory threshold was 20 s (Range 0–120). The median % of correct attempts at pointing was 83.33% (Range 33–100%), at inhibitory control was 87.5 % (Range 0–100%) and at detour was 44% (Range 0–100%). Fractional lifespan was correlated with CADES score (ρ = 0.641, *p* ≤ 0.001), sustained gaze (ρ = −0.521, *p* ≤ 0.001), memory (ρ = −0.547, *p* = 0.001), inhibitory control (ρ = −0.480, *p* ≤ 0.001) and detour (ρ = −0.493, *p* ≤ 0.001). No significant correlation was found between fractional lifespan and score in the pointing test (ρ = −0.275, *p* = 0.085).

Table 1 provides the correlation between gait speed results and the performance in cognitive testing. Gait speed off leash was positively correlated with sustained gaze, memory, and detour. Additionally, gait speed off leash was correlated with CADES score and inhibitory control test.

**Table 1:** Correlation between gait speed and cognitive performance in senior dogs.

**Cognitive performance**	**Gait speed on leash**		**Gait speed off leash**	
	**Spearman's** ρ	***p*** **-value**	**Spearman's** ρ	***p*** **-value**
CADES	−0.109	0.457	−0.289	0.046^*^
Sustained gaze	−0.053	0.716	0.438	0.002^*^
Pointing	0.263	0.100	0.379	0.016^*^
Memory	0.075	0.616	0.307	0.005^*^
Inhibitory Ctrl	0.213	0.146	0.388	0.006^*^
Detour	0.037	0.806	0.347	0.018^*^

Results with a *p* < 0.05 are indicated with an ^*^.

As shown in Table 2, after adjustment for fractional lifespan, gait speed off leash remained significantly associated with performance in the sustained gaze, memory and pointing tests suggesting dogs who moved faster also performed better on cognitive testing independent of their life stage. Additionally, there was a significant effect of the interaction between gait speed off leash and fractional lifespan on CADES score, sustained gaze and inhibitory control tasks.

**Table 2:** Multivariate analysis evaluating the relationship between gait speed off leash and cognitive performance including fractional lifespan as a covariate.

		**Intercept**	**Fractional lifespan**		**Gait speed off leash**		**Fractional lifespan** × **Gait speed off leash**	
		***p***	***Std B***	***p***	***Std B***	***p***	***Std B***	***p***
CADES	R^2^ = 0.442	0.013^*^	0.598	< 0.001^*^	0.023	0.854	−0.320	0.007^*^
Sustained gaze	R^2^ = 0.327	0.001^*^	−0.284	0.045^*^	0.316	0.026^*^	0.277	0.030^*^
Pointing	R^2^ = 0.268	< 0.001^*^	−0.214	0.173	0.316	0.048^*^	0.289	0.051
Working memory	R^2^ = 0.311	0.019^*^	−0.285	0.049^*^	0.437	0.017^*^	0.217	0.098
Inhibitory control	R^2^ = 0.365	< 0.001^*^	−0.350	0.012^*^	0.263	0.056	0.320	0.011^*^
Detour	R^2^ = 0.153	0.009^*^	−0.320	0.048^*^	0.105	0.509	0.135	0.346

Non-normally distributed variables were transformed by log (x + 1) to achieve normality. Results with a p < 0.05 are indicated with an ^*^.

### The relationship between pain and speed

**Figure 2:**
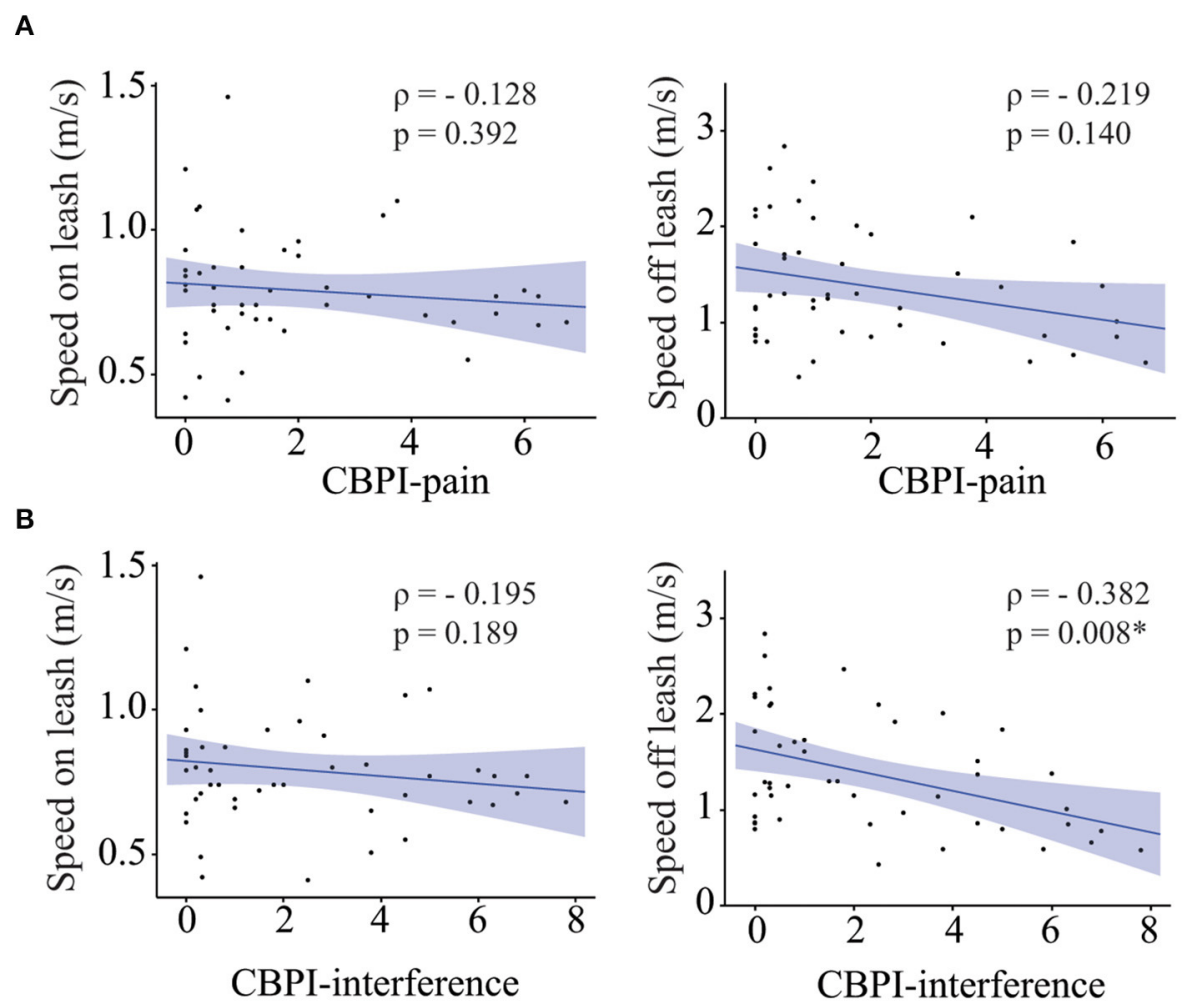
Role of pain on gait speed. Correlation between CBPI-pain scores **(A)** [and CBPI-interference scores **(B)**] and gait speed on leash (left), gait speed off leash (right). Asterisks indicate significant differences.

Owners of 47 of the 49 senior dogs included in this study completed the CBPI pain questionnaire. The median score was 1 (Range 0–6.75) for the CBPI pain severity subscale and 1.5 (Range 0–7.8) for the CBPI pain interference subscale. No correlation was found between the CBPI pain severity subscale and gait speed on leash, or gait speed off leash (Figure 2A) while CBPI pain interference subscale was negatively correlated with gait speed off leash, but not with gait speed on leash (Figure 2B). However, when we adjusted for fractional lifespan, these associations did not remain statistically significant (fractional lifespan *p* = 0.002, CBPI pain interference *p* = 0.122 and fractional lifespan × CBPI pain interference *p* = 0.573).

Additionally, complete orthopedic examination was performed in 47 of the 49 dogs. The examination was aborted in two dogs who behaved aggressively. Median score for total joint pain was 5 (range 0–17). There was no correlation between total joint pain and gait speed on leash, or off leash (ρ = 0.115, *p* = 0.450 and ρ = −0.235, *p* = 0.120 respectively).

## Discussion

In people, gait speed has been proven to be a reliable measure of physical function and to be associated with frailty, disability, mortality, and cognitive decline (7, 9, 28). In this work we demonstrated that dogs show age-related changes in gait speed and that gait speed is associated with cognitive performance, independent of pain scores. We measured gait speed on leash while being walked by a handler, and gait speed off leash by allowing the dog to walk, trot or run toward a treat. We found that taller dogs were significantly faster on leash (in the group of senior dogs) and off leash (in the group of young dogs). Interestingly, in senior dogs, height was not associated with gait speed off leash. This could suggest that in senior dogs age-related changes might have a much stronger effect than height. We posit that gait speed off leash may capture the effects of both physical ability and motivation (food motivation in particular). In people, a role for motivation in gait speed is also suspected, as apathy (reduction in goal directed activity) is associated with slower walking speed and with risk of dementia (29).

### Role of age

Age was correlated with gait speed off leash in the group of senior dogs. These results agree with a previous study by Morgan et al. (15); however, those authors also found age-related variations in gait speed on leash. A potential reason for this difference is that in the previous study, the experimenters trotted the dog instead of walking them. Additionally, they measured gait speed over a longer distance (10 m instead of 5) and longer distances might be able to capture the effect of exhaustion in older dogs more easily. Nevertheless, their study also found that the strength of the correlation with age was higher when gait speed was measured off leash than on leash. It is important to take into consideration that speed on leash might be also influenced by the experimenter; while the dog was allowed to set the pace, pet dogs tend to match the speed of the handler when walking on leash (30). In addition to this, our study shows that gait speed on leash has a lower test-retest reliability than gait speed off leash.

Noteworthy in our study, the association between speed and age was only seen in the group of senior dogs, but no association was found in the group of adult dogs (defined as from 1 year old to 75% of expected lifespan) (20). These results demonstrate that in dogs, gait speed decreases with age in a non-linear fashion. The same has been shown to occur in humans and mice in whom decline in gait speed starts to occur at approximately 70% of their expected lifespan (31). Hence, our results further support the dog as a translational model of aging.

We found that gait speed off leash had a stronger association with fractional lifespan (the life stage) than with the chronological number of years. This is particularly relevant in dogs, since their lifespan is highly dependent on body size, and this species is characterized by a huge size diversity (32, 33). Scientists have been investigating the impact of aging rates in specific health domains in different breeds of dog (34–36). In this regard two hypotheses have been proposed: the compression hypothesis, which states that age-related changes occur earlier in larger dogs than in smaller dogs and are related to life stage rather than chronological age, and the truncation hypothesis, which purports that aging is related to chronological age rather than lifestage, thus larger dogs frequently die prior to developing certain age-related changes (37). Current evidence suggests that not all health-domains behave the same way; some studies have shown that larger dogs age at an accelerated rate (38, 39), however, Watowich et al. (37) demonstrated that age-related changes in cognition are largely independent of the expected lifespan (37). Our study suggests that variations in gait speed may fit best with the compression hypothesis and are related to lifestage rather than chronological age. Similarly, Morgan et al. (15) showed that dogs older than 75% of their expected lifespan had slower gait speeds than younger dogs (15).

### Association between gait speed and cognitive performance

Gait speed off leash was correlated with several domains of cognitive performance. For instance, it was negatively correlated with CADES score and positively correlated with performance in all the cognitive tests, showing that the speed at which dogs are willing to move in order to obtain a food reward is associated with specific domains of cognition. When correcting for fractional lifespan, gait speed off leash remained correlated with sustained gaze and memory; both tasks that require sustained attention. Studies in humans have shown similar results; gait speed was more strongly associated with attention than with other measures of executive function (40, 41). However, another study found that gait slowing was associated with poorer performance in several different cognitive tasks, and they propose that there is no specific domain in cognitive decline that accounts for the reduction in gait speed (42). As we were measuring gait speed toward a treat, we consider that we are not measuring just the physical ability of the dogs, but also their motivation. Future studies should evaluate the relationship between motivation and different cognitive domains in dogs, and if this could explain why speed off leash are correlated with only some of the cognitive domains.

Studies in people have shown that specific areas of the brain are greatly atrophied in those with declines in memory and gait speed (known as dual decliners) in comparison with people with decline in only one domain (41). Some of these areas are the frontal gyrus, cerebellum, precuneus and thalamus. The last two are critical for sensorimotor integration (43). While the cerebellum is mainly known for its role in control and integration of motor activity, it also engages in cognitive processing (44). In fact, in people, cerebellar lesions can impair cognitive function (45) and neuroimaging studies have demonstrated cerebellar activation in executive and working-memory processes (46). In dogs with CCDS brain atrophy, reduction of the inter-thalamic adhesion and hippocampal volume has been demonstrated (47, 48). Moreover, aging is associated with a loss of Purkinje and granular cells and dogs with CCDS showed a further reduction in Purkinje cells (49) than age-matched healthy dogs. Further studies are needed to determine whether dogs with dual decline (in gait speed and cognition) are characterized by specific brain changes on MRI.

We did find an association between gait speed and CADES score but this association was lost when correcting for fractional lifespan. This could be explained by the fact that while the CADES questionnaire has been validated (19), it relies on the sensitivity of the owner to detect behavioral changes. Moreover, this questionnaire evaluates cognitive decline by asking about several different behavioral domains, without looking at particular domains of cognition.

### Association between gait speed and pain

Finally, since prevalence of chronic pain conditions such as osteoarthritis increase with age in dogs (50), we wanted to investigate the relationship between pain and gait speed. Regarding this, a recent study has shown that according to the owner's perception, dogs with osteoarthritis walk slower than normal physically sound dogs (51). However, in our study, neither the CBPI pain severity subscale nor total joint pain were correlated with speed on or off leash. We did find an association between the CBPI pain interference subscale and gait speed off leash, but this association was lost when correcting for fractional lifespan. However, these results do not rule out the role of pain in declining speed in dogs, since our population of dogs were not particularly painful, (i.e., the highest joint pain score was 17, which is only 27% of the highest possible score for this scale). Further studies in dogs with diagnosed osteoarthritis are warranted.

Our study had some limitations. Firstly, gait speed measurements and cognitive tests were conducted without the presence of the dogs' owners, who serve as their attachment figure. This may have influenced the behavior and performance of dogs with separation anxiety (52). We did not use any clinical metrology instrument to measure separation anxiety in these dogs, however, we excluded dogs that displayed signs of anxiety and avoided approaching the experimenters for treats, even though we did not use any clinical metrology instrument to measure separation anxiety. Additionally, while the presence of the owner may be beneficial for some specific dogs, it could potentially interfere with testing results (53) or cause dogs to exhibit more aggression (54). Nonetheless, we ensured that the experimental conditions were consistent within and across dogs, which we believe is more crucial than the presence or absence of the owner during testing. Secondly, since this was an exploratory study, we did not adjust for multiple comparisons. Therefore, future studies should investigate the correlation between gait speed off leash and various cognitive domains in greater detail and with a larger number of dogs to confirm our findings.

## Conclusion

In conclusion, our study shows that gait speed off leash is a relatively easy variable to measure in clinical settings and is correlated with fractional lifespan and cognitive performance in dogs. Moreover, gait speed off leash seems to be a better measure than gait speed on leash as an indicator of age-related deterioration and cognitive decline. This simple and non-invasive measurement is an impactful addition to assessment of senior and geriatric dogs.

## Data availability statement

The original contributions presented in the study are included in the article/Supplementary material, further inquiries can be directed to the corresponding author.

## Ethics statement

The animal study was reviewed and approved by the Institutional Animal Use and Care Committe, NCSU. Written informed consent was obtained from the individual(s) for the publication of any identifiable images or data included in this article.

## Author contributions

AM: conceived and designed work, acquired and interpreted data, and wrote manuscript. MK and BC: acquired and interpreted data, and reviewed manuscript. GF and WP: designed work, acquired and interpreted data and reviewed manuscript. MG: designed work, reviewed and interpreted data, and reviewed manuscript. NO: conceived and designed work, reviewed and interpreted data, and reviewed manuscript. All authors contributed to the article and approved the submitted version.

## References

[1] McKenzie BA Chen FL Gruen ME Olby NJ. Canine geriatric syndrome: a framework for advancing research in veterinary geroscience. Front Vet Sci. (2022) 9:853743. 10.3389/fvets.2022.853743 35529834 PMC9069128

[2] McKenzie BA Chen FL. Assessment and management of declining physical function in aging dogs. Top Companion Anim Med. (2022) 51:100732. 10.1016/j.tcam.2022.100732 36273752

[3] Booth F Weeden S Tseng B. Effect of aging on human skeletal muscle and motor function. Med. Sci Sports Exercise. (1994) 26:556–60. 10.1249/00005768-199405000-00006 8007802

[4] Peel NM Kuys SS Klein K. Gait speed as a measure in geriatric assessment in clinical settings: a systematic review. J Gerontol A Biol Sci Med Sci. (2013) 68:39–46. 10.1093/gerona/gls174 22923430

[5] Mondino A Wagner G Russell K Lobaton E Griffith E Gruen M. Static posturography as a novel measure of the effects of aging on postural control in dogs. PLoS ONE. (2022) 17:e0268390. 10.1371/journal.pone.0268390 35802714 PMC9269968

[6] Stuck AK Bachmann M Fullemann P Josephson KR Stuck AE. Effect of testing procedures on gait speed measurement: a systematic review. PLoS ONE. (2020) 15:e0234200. 10.1371/journal.pone.0234200 32479543 PMC7263604

[7] Fritz S Lusardi M. White paper: “walking speed: the sixth vital sign”. J. Geriatr. Phys. Ther. (2009) 2:2–5. 10.1519/00139143-200932020-00002 20039582

[8] Hardy SE Perera S Roumani YF Chandler JM Studenski SA. Improvement in usual gait speed predicts better survival in older adults. J Am Geriatr Soc. (2007) 55:1727–34. 10.1111/j.1532-5415.2007.01413.x 17916121

[9] Buracchio T Dodge H Howieson D Wasserman D Kaye J. The trajectory of gait speed preceding mild cognitive impairment. Arch Neurol. (2010) 67:980–6. 10.1001/archneurol.2010.159 20697049 PMC2921227

[10] Grande G Triolo F Nuara A Welmer AK Fratiglioni L Vetrano DL. Measuring gait speed to better identify prodromal dementia. Exp Gerontol. (2019) 124:110625. 10.1016/j.exger.2019.05.014 31173841

[11] Dumurgier J Artaud F Touraine C Rouaud O Tavernier B Dufouil C. Gait speed and decline in gait speed as predictors of incident dementia. J Gerontol A Biol Sci Med Sci. (2017) 72:655–61. 10.1093/gerona/glw110 27302701

[12] Meiner Z Ayers E Verghese J. Motoric cognitive risk syndrome: a risk factor for cognitive impairment and dementia in different populations. Ann Geriatr Med Res. (2020) 24:3–14. 10.4235/agmr.20.0001 32743316 PMC7370775

[13] Head E A. Canine model of human aging and Alzheimer's disease. Biochim Biophys Acta. (2013) 1832:1384–9. 10.1016/j.bbadis.2013.03.016 23528711 PMC3937962

[14] Lorke M Willen M Lucas K Beyerbach M Wefstaedt P Murua Escobar H. Comparative kinematic gait analysis in young and old beagle dogs. J Vet Sci. (2017) 18:521–30. 10.4142/jvs.2017.18.4.521 28385001 PMC5746446

[15] Morgan E Heseltine JC Levine GJ Promislow D Creevy KE. Evaluation of a low-technology system to obtain morphological and mobility trial measurements in dogs and investigation of potential predictors of canine mobility. AJVR. (2019) 80:670–9. 10.2460/ajvr.80.7.670 31246119 PMC7311064

[16] Fefer G Panek WK Khan MZ Singer M Westermeyer H Mowat FM. Use of cognitive testing, questionnaires, and plasma biomarkers to quantify cognitive impairment in an aging pet dog population. J Alzheimers Dis. (2022). 10.3233/JAD-215562 35431246 PMC9177825

[17] Azkona G Garcia-Belenguer S Chacon G Rosado B Leon M Palacio J. Prevalence and risk factors of behavioural changes associated with age-related cognitive impairment in geriatric dogs. J Small Anim Pract. (2009) 50:87–91. 10.1111/j.1748-5827.2008.00718.x 19200264

[18] Mihevc SP Majdic G. Canine cognitive dysfunction and alzheimer's disease - two facets of the same disease? Front Neurosci. (2019) 13:604. 10.3389/fnins.2019.00604 31249505 PMC6582309

[19] Madari A Farbakova J Katina S Smolek T Novak P Weissova T. Assessment of severity and progression of canine cognitive dysfunction syndrome using the canine dementia scale (Cades). Appl Anim Behav Sci. (2015) 171:138–45. 10.1016/j.applanim.2015.08.034

[20] Creevy KE Grady J Little SE Moore GE Strickler BG Thompson S. 2019 Aaha canine life stage guidelines. J Am Anim Hosp Assoc. (2019) 55:267–90. 10.5326/JAAHA-MS-6999 31622127

[21] Greer KA Canterberry SC Murphy KE. Statistical analysis regarding the effects of height and weight on life span of the domestic dog. Res Vet Sci. (2007) 82:208–14. 10.1016/j.rvsc.2006.06.005 16919689

[22] Panek WK Murdoch DM Gruen ME Mowat FM Marek RD Olby NJ. Plasma amyloid beta concentrations in aged and cognitively impaired pet dogs. Mol Neurobiol. (2021) 58:483–9. 10.1007/s12035-020-02140-9 32970242 PMC7855498

[23] Panek WK Gruen ME Murdoch DM Marek RD Stachel AF Mowat FM. Plasma neurofilament light chain as a translational biomarker of aging and neurodegeneration in dogs. Mol Neurobiol. (2020) 57:3143–9. 10.1007/s12035-020-01951-0 32472519 PMC7529326

[24] Cimino Brown D Boston RC Coyne JC Farrar JT. Ability of the canine brief pain inventory to detect response to treatment in dogs with osteoarthritis. J Am Vet Med Assoc. (2008) 15:1278–83. 10.2460/javma.233.8.1278 19180716 PMC2896492

[25] Knazovicky D Helgeson ES Case B Gruen ME Maixner W Lascelles BDX. Widespread somatosensory sensitivity in naturally occurring canine model of osteoarthritis. Pain. (2016) 157:1325–32. 10.1097/j.pain.0000000000000521 26901805 PMC4866583

[26] Koo TK Li MY A. Guideline of selecting and reporting intraclass correlation coefficients for reliability research. J Chiropr Med. (2016) 15:155–63. 10.1016/j.jcm.2016.02.012 27330520 PMC4913118

[27] Barnett MJ Doroudgar S Khosraviani V Ip EJ. Multiple comparisons: to compare or not to compare, that is the question. Res Social Adm Pharm. (2022) 18:2331–4. 10.1016/j.sapharm.2021.07.006 34274218

[28] Studenski SA Perera S Patel K Rosano C Faulkner K Inzitari M. Gait speed and survival in older adults. J Am Med Assoc. (2011) 5:50–8. 10.1001/jama.2010.1923 21205966 PMC3080184

[29] Ceide ME Warhit A Ayers EI Kennedy G Verghese J. Apathy and the risk of predementia syndromes in community-dwelling older adults. J Gerontol B Psychol Sci Soc Sci. (2020) 75:1443–50. 10.1093/geronb/gbaa063 32374839 PMC7424283

[30] Pfau T Roberts TWM Weller R. Monitoring of Excercise in Dogs and Handlers Using Gps. Brussels: International Society of Biomechanics (2011).

[31] Bair WN Petr M Alfaras I Mitchell SJ Bernier M Ferrucci L. Of Aging mice and men: gait speed decline is a translatable trait, with species-specific underlying properties. J Gerontol A Biol Sci Med Sci. (2019) 74:1413–6. 10.1093/gerona/glz015 30649206 PMC6696716

[32] Wayne RK Ostrander EA. Origin, genetic diversity, and genome structure of the domestic dog. BioEssays. (1999) 21:247–57. 10.1002/(SICI)1521-1878(199903)21:3&lt;247::AID-BIES9&gt;3.0.CO;2-Z 10333734

[33] Yordy J Kraus C Hayward JJ White ME Shannon LM Creevy KE. Body size, inbreeding, and lifespan in domestic dogs. Conserv Genet. (2020) 21:137–48. 10.1007/s10592-019-01240-x 32607099 PMC7326369

[34] Creevy KE Austad SN Hoffman JM O'Neill DG Promislow DE. The Companion dog as a model for the longevity dividend. Cold Spring Harb Perspect Med. (2016) 6:a026633. 10.1101/cshperspect.a026633 26729759 PMC4691800

[35] Fleming JM Creevy KE Promislow DE. Mortality in north american dogs from 1984 to 2004: an investigation into age-, size-, and breed-related causes of death. J Vet Intern Med. (2011) 25:187–98. 10.1111/j.1939-1676.2011.0695.x 21352376

[36] Selman C Nussey DH Monaghan P. Ageing: it's a dog's life. Curr Biol. (2013) 23:R451–3. 10.1016/j.cub.2013.04.005 23701689

[37] Watowich MM MacLean EL Hare B Call J Kaminski J Miklosi A. Age influences domestic dog cognitive performance independent of average breed lifespan. Anim Cogn. (2020) 23:795–805. 10.1007/s10071-020-01385-0 32356029 PMC7384235

[38] Fan R Olbrithc G Baker X Hou C. Birth mass is the key to understanding the negative correlation between lifespan and body size in dogs. Aging. (2016) 8:3209–21. 10.18632/aging.101081 27956710 PMC5270664

[39] Kraus C Pavard S Promislow DE. The size-life span trade-off decomposed: why large dogs die young. Am Nat. (2013) 181:492–505. 10.1086/669665 23535614

[40] Park H Aul C DeGutis J Lo OY Poole VN McGlinchey R. Evidence for a specific association between sustained attention and gait speed in middle-to-older-aged adults. Front Aging Neurosci. (2021) 13:703434. 10.3389/fnagi.2021.703434 34290601 PMC8289388

[41] Tian Q Studenski SA Montero-Odasso M Davatzikos C Resnick SM Ferrucci L. Cognitive and neuroimaging profiles of older adults with dual decline in memory and gait speed. Neurobiol Aging. (2021) 97:49–55. 10.1016/j.neurobiolaging.2020.10.002 33152563

[42] Watson NL Rosano C Boudreau RM Simonsick EM Ferrucci L Sutton-Tyrrell K. Executive function, memory, and gait speed decline in well-functioning older adults. J Gerontol A Biol Sci Med Sci. (2010) 65:1093–100. 10.1093/gerona/glq111 20581339 PMC2949334

[43] Tian Q Resnick SM Mielke MM Yaffe K Launer LJ Jonsson PV. Association of dual decline in memory and gait speed with risk for dementia among adults older than 60 years: a multicohort individual-level meta-analysis. JAMA Netw Open. (2020) 3:e1921636. 10.1001/jamanetworkopen.2019.21636 32083691 PMC7043189

[44] Won J Callow DD Purcell JJ Smith JC. Differential associations of regional cerebellar volume with gait speed and working memory. Sci Rep. (2022) 12:2355. 10.1038/s41598-022-06180-0 35149757 PMC8837608

[45] Bolcekova E Mojzes M Van Tran Q Kukal J Ostry S Kulistak P. Cognitive impairment in cerebellar lesions: a logit model based on neuropsychological testing. Cerebellum Ataxias. (2017) 4:13. 10.1186/s40673-017-0071-9 28775852 PMC5534033

[46] Ben-Yehudah G Guediche S Fiez JA. Cerebellar contributions to verbal working memory: beyond cognitive theory. Cerebellum. (2007) 6:193–201. 10.1080/14734220701286195 17786815

[47] Dewey CW Rishniw M Johnson PJ Davies ES Sackman JJ O'Donnell M. Interthalamic adhesion size in aging dogs with presumptive spontaneous brain microhemorrhages: a comparative retrospective mri study of dogs with and without evidence of canine cognitive dysfunction. PeerJ. (2020) 8:e9012. 10.7717/peerj.9012 32322448 PMC7161569

[48] Dewey CW Rishniw M Johnson PJ Platt S Robinson K Sackman J. Canine cognitive dysfunction patients have reduced total hippocampal volume compared with aging control dogs: a comparative magnetic resonance imaging study. Open Vet J. (2021) 10:438–42. 10.4314/ovj.v10i4.11 33614439 PMC7830179

[49] Pugliese M Gangitano C Ceccariglia S Carrasco JL Del Fa A Rodriguez MJ. Canine cognitive dysfunction and the cerebellum: acetylcholinesterase reduction, neuronal and glial changes. Brain Res. (2007) 1139:85–94. 10.1016/j.brainres.2006.12.090 17292335

[50] Anderson KL Zulch H O'Neill DG Meeson RL Collins LM. Risk factors for canine osteoarthritis and its predisposing arthropathies: a systematic review. Front Vet Sci. (2020) 7:220. 10.3389/fvets.2020.00220 32411739 PMC7198754

[51] Belshaw Z Dean R Asher L. Slower, shorter, sadder: a qualitative study exploring how dog walks change when the canine participant develops osteoarthritis. BMC Vet Res. (2020) 16:85. 10.1186/s12917-020-02293-8 32156275 PMC7063782

[52] Konok V Dóka A Miklósi Á. The behavior of the domestic dog (*Canis familiaris*) during separation from and reunion with the owner: a questionnaire and an experimental study. Appl Anim Behav Sci. (2011) 135:300–8. 10.1016/j.applanim.2011.10.011

[53] Miklósi Á. Methodological Issues in the Behavioural Study of the Dog. Dog Behaviour, Evolution, and Cognition. New York, NY: Oxford University Press (2014). p. 39-67.

[54] Kis A Klausz B Persa E Miklosi A Gacsi M. Timing and presence of an attachment person affect sensitivity of aggression tests in shelter dogs. Veterinary Record. (2014) 174:196. 10.1136/vr.101955 24482210

